# Assessment of drug use in primary health centers in Lagos State, Nigeria

**DOI:** 10.11604/pamj.2022.43.58.36231

**Published:** 2022-10-07

**Authors:** Shakirat Adeshiyan Adeosun, Arinola Eniola Joda, Roseline Iberi Aderemi-Williams, Olubukola Olusola Oyetunde

**Affiliations:** 1 Department of Clinical Pharmacy and Biopharmacy, Faculty of Pharmacy, University of Lagos, Idiaraba, Lagos State, Nigeria

**Keywords:** Drug use, Lagos State, Nigeria, primary health centres

## Abstract

**Introduction:**

rational drug use prevents wastage of resources, loss of confidence in healthcare system and drug-related morbidity and mortality. This study aims to assess drug use in Primary Health Centers (PHCs) in Lagos State, Nigeria using the World Health Organization in collaboration with the International Network of Rational Use of Drugs core drug use indicators.

**Methods:**

the study was conducted between February to October 2021 as a comparative observational survey of selected PHCs. It included a retrospective and prospective cross-sectional design for prescribing and patient care indicators assessing 2640 prescriptions and clients respectively. Data were analyzed and presented as frequency with percentage or mean with standard deviation, as applicable. The performances of the types of PHCs were compared using two-sample t-test. A 2-tailed p-value < 0.05 was considered statistically significant.

**Results:**

average number of drugs per prescription, drugs prescribed by the generic name, percentage of encounters with prescribed antibiotics and injections were 3.6 ± 0.9%, 76.5 ± 18.5%, 63.3 ± 19.1% and 21.1 ± 24.1% respectively with no significance difference between the comprehensive and basic PHCs. For all the facilities, the average consultation and dispensing times were 10.5 ± 6.0 minutes, 244.9 ± 179.2 seconds respectively. In this study, the percentage of patients’ knowledge of the correct dosage was 72.4 ± 38.3%. There is statistically significant difference in availability of key drugs in stock between the comprehensive and basic PHCs (p-value 0.0001).

**Conclusion:**

irrational drug use practices exist in comprehensive and basic PHCs. There is a need to implement interventions aimed at strengthening good prescribing and patient-care practices across the PHCs in Lagos State.

## Introduction

A valuable resource that is an indicator of health care quality worldwide is drugs [1]. An essential element in achieving quality health and medical care for the patients and the community is the rational use of drugs [2]. About 50% of patients adhere to their medications worldwide. In developing and transitional countries, about 50% of all dispensing events are inadequate in instructing patients and/or labeling dispensed medicines [3]. Wastage of resources, loss of confidence in the healthcare system, and drug-related morbidity and mortality are associated with irrational drug use [4,5]. Polypharmacy, self-medication, inappropriate use of antibiotics, overuse of injectables, and prescribing of medicines that are not according to clinical practice guidelines are the most common expression of irrational medicine use [5-7].

The Nigerian National Drug Policy stipulates that essential medicines are selected based on the prevailing disease conditions and rational drug use is attained when there is rational prescribing of drugs in generic or non-proprietary names and from an Essential Drugs List (EDL) [8]. The World Health Organization (WHO), in collaboration with the International Network of Rational Use of Drugs (INRUD), developed a set of indicators to measure the performance of healthcare facilities. These indicators are core drug use indicators and comprise prescribing, patient care, and facility-specific indicators [9]. Several studies [10,11] showed that an initial limitation to the use of the WHO/INRUD indicators was the lack of reference standard values which are benchmarks for comparison when assessing rational drug use and evaluating interventional and supervisory efforts. Zhang and Zhi [12] however developed an Index of Rational Drug Use (IRDU) to address this limitation which has been successfully employed for comprehensive evaluation [6,13].

Many studies have investigated drug use practices in health facilities in Nigeria. Studies within Nigeria [14-16] have assessed prescribing practices in selected PHCs. Other studies evaluated prescribing practices in Nigeria´s selected secondary [17-19] and tertiary [20-23] health facilities. Prescribing practices have also been evaluated in selected primary and secondary health facilities [24] and in selected primary, secondary, and tertiary healthcare facilities in Nigeria [25]. However, most of the previous studies focused on prescribing practices. Hence, there is a paucity of information on patient care and facility-specific factors that affect rational drug use in Nigeria. Moreover, few studies have assessed drug use practices at the primary level of healthcare in Nigeria. A bibliometric review of studies on rational drug use in Nigeria between 1985 and 2013 shows that less than 20% of the studies were conducted in PHCs and less than five studies on drug use practices were conducted at PHCs in Lagos State [26]. In addition, there are no data on the comparative performance of PHCs in Lagos State based on IRDU.

There is a need for comprehensive data on the current state of drug use practices at the primary level of healthcare in Lagos State. The design of the present study was to address these identified gaps. This study aimed to assess drug use in PHCs in Lagos State, Nigeria using the WHO/INRUD core drug use indicators and the index of rational drug use.

## Methods

**Study area and period:** the study was carried out in selected PHCs in Lagos State. Lagos State is made of five administrative divisions, namely: Ikeja, Badagry, Ikorodu, Lagos, and Epe [27]. At the time of the study, there were 309 functional PHCs in Lagos State. The two categories of PHCs in Lagos State are comprehensive PHCs which operate on a twenty-four-hour daily basis and the basic PHCs which operate on an eight-hour daily basis [28]. The study was conducted between February 2021 and October 2021.

**Study design:** this study involved a comparative observational survey in selected PHCs in Lagos State, Nigeria. The observational study included a retrospective cross-sectional design for prescribing indicators; and a prospective cross-sectional design for patient care and facility specific indicators using WHO methodology.

**Inclusion criteria:** i) basic primary health centers; ii) comprehensive primary health centers.

**Exclusion criteria:** i) health posts.

**Sample size determination and sampling technique:** WHO (1993) recommends a sample size of at least 600 encounters or with a greater number if possible for a cross sectional study. There should be a minimum of 30 encounters per facility [9]. A total of eighty-eight PHCs were selected following the two staged stratified random sampling technique to determine the sample size. For the first stage, they were stratified into comprehensive and basic PHCs. Primary health centers were selected from each secondary stratum by simple random sampling, using the list of eligible PHCs in each stratum as the sampling frame. Four comprehensive PHCs each were equally allocated across the five administrative divisions of Lagos State making a total of twenty comprehensive PHCs. Sixty-eight basic PHCs were also proportionally selected respectively across the five administrative divisions of Lagos State. Sampling frames were constructed from 277 eligible PHCs that met the inclusion criteria out of the total 309 PHCs in Lagos State at the time of this study. Computer-generated (MS Excel 2016) random numbers were used. In each of the selected PHCs, 30 prescribing encounters were randomly sampled from prescription sheets. In addition, 30 patients per facility (aged 18 and older) were consecutively recruited by convenient sampling into the study for data on patient care indicators. Prior to the main survey, a pilot study was also conducted involving 10 patients each from 2 different PHCs. Patients were interviewed to estimate the time required to collect the variables from each patient, ensure availability of the necessary data, edit the data collection tool as required and remove ambiguity while ensuring clarity of respondents' opinion. The two PHCs selected for the pilot study were not included as part of the main study.

**Sample size determination:** the sample size was computed using the following formula [29]:


$$n=\frac{Z_{\alpha }^{2}*S^{2}}{d^{2}}$$

where Z_α_= 1.96 (standard normal deviate at 95% confidence level); S = 0.5 (standard deviation of the average drugs/encounter); and d = 0.15 (level of precision) [22,30].


$$\begin{array}{ll} n= & \frac{1.96^{2}\times 0.5^{2}}{0.15^{2}}=\frac{0.9604}{0.0225}\\ n= & 42.6844 \end{array}$$

The effective sample size computed was 86 PHCs, after adjusting for a design effect of 2.0.

n *_eff_*= 42.6844 x 2.0 = 85.3688; n *_eff_*≌86

**Health facility indicators:** a total of 19 essential medicines (Annex 1) which are used to treat the most prevalent conditions were chosen to be assessed as key drugs for testing drug availability in the basic and comprehensive PHCs in line with the recommendations (minimum of 15 key medicines in each facility) [9].

**Patient care indicators:** a minimum of 30 encounters per facility were observed when data was collected using the patient care indicator forms as recommended by WHO [9]. A total of 2640 randomly selected samples of respondents were evaluated across the 88 PHCs using the patient care indicator form.

**Prescribing indicators:** as recommended by WHO, a minimum of 30 prescribing encounters were considered for assessment [9]. A total of 2640 prescriptions from 88 primary health care centers were assessed.

**Data collection instrument, measurements, and techniques:** the drug use indicators (patient care, prescriber and health facility), WHO/INRUD detailed indicators encounter form are standardized data collection tools used for the study. The drug labelling form and patient´s knowledge form were designed for the purpose of collecting additional data. The evaluation of the core drug use indicators was according to the WHO/ International Network of Rational Use of Drugs (INRUD) guidelines [9,31].

**Health facility indicators measurement:** the WHO health facility indicators were used in this research using the health facility indicator checklist to collect data relating to availability of essential medicines [9,31]. The health facility indicators that were assessed included the availability of Essential Medicines list and percentage of key [9] drugs in stock across the 88 PHCs. Data was collected prospectively.

**Patient care indicators measurement:** trained data collectors interviewed patients focusing on knowledge about dispensed medications (drug name, dosage, and drug strength) thus assessing the effectiveness of counselling the patients. Observation of the adequacy of labelling of medicine package or drug envelope was also noted. The data collectors ensured clinic flow was not disrupted and the same patients were followed through when observing the consultation and dispensing process. Patients were interviewed away from the main clinic to assess their knowledge base of medicines dispensed. Data was collected prospectively.

**Prescribing indicators measurement:** data on prescribing indicators were collated retrospectively from randomly selected prescriptions and filled in prescribing indicator form in line with WHO methodology [9].

**Calculation of the measurement of the core drug use indicators:** formula for measurement of drug use indicators were obtained using the WHO guidelines [9].

### Prescribing indicators


$$\text{Average number of drugs per encounter}=\frac{\text{total number of drugs}}{\text{total number of encounters}}$$

$$\text{Percentage of encounters with an antiblotic prescribed=}\frac{\text{total number of eneric drugs prescribed}}{\text{total number of drug prescribed}}\times 100\%$$

$$\text{Percentage of encounters with an antibiotic prescribed}=\frac{\text{total number of patients who received on or more antibiotics}}{\text{total number of encounters}}\times 100\%$$

$$\text{Percentage of encounters with an injection prescribed}=\frac{\text{total number of patients who received on or more injections}}{\text{total number of encounters}}\times 100\%$$

$$\text{Percentage of drugs prescribed from essential drus list (EDL) or formulary}=\frac{\text{total number of EDL drugs prescribed}}{\text{total number of drugs prescribed}}\times 100\%$$

### Patient care indicators


$$\text{Average consultation time}=\frac{\text{sum of all consulting times}}{\text{total number of cases}}$$

$$\text{Average dispensing time}=\frac{\text{sum of all despensing times}}{\text{total number of cases}}$$

$$\text{Percentage of drugs actually dispensed}=\frac{\text{total number of drugs actually dispensed}}{\text{total number of drugs prescribed}}\times 100\%$$

$$\text{Percentage of drugs adequately labeled}=\frac{\text{sum of the number of drugs with adequate labels for each patient}}{\text{total number of drugs dispensed}}\times 100\%$$

$$\text{Percentage knowledge of correct dosage}=\frac{\text{sum of patients who can correctly report all their drug dosage}}{\text{total number of patients interviewed}}\times 100\%$$

**Facility-specific indicators:** availability of copy of EDL or formulary. This indicator is scored either 1 (yes) or 0 (no), for the facility as a whole.


$$\text{Availability of key drugs}=\frac{\text{number of key drugs in stock}}{\text{total number of key drugs surveyed}}\times 100\%$$

**Data management and statistical analysis:** data collected, reviewed and verified were entered in MS Excel (2016) and statistically analyzed in IBM SPSS (version 23). Data were summarized using frequency with percentage or mean with standard deviation, as applicable. The performances of PHCs on drug use indicators were compared across the type of functional PHCs in Lagos State for statistically significant difference using two-sample t-test. A 2-tailed p-value < 0.05 was considered statistically significant.

**Ethical considerations:** ethical approval with health research assigned number ADM/DCST/HREC/APP/2257 was obtained from the Health Research Ethics Committee, Lagos University Teaching Hospital. The Lagos State Government through the Lagos State Primary Health Care Board also granted approval to carry out the study. Verbal consent from each patient to participate in the study was obtained before the interview.

## Results

A total of 2640 prescriptions were encountered in the selected 88 PHCs in Lagos State.

**Drug prescribing indicators:** average number of drugs prescribed per prescription were 3.6 ± 0.9%, 3.8 ± 0.9% and 3.5 ± 0.9% for all the PHCs, comprehensive PHCs and basic PHCs respectively. For all the PHCs, the drugs prescribed by the generic name was 76.5 ± 18.5%. The difference among the PHCs was not statistically significant for all prescribing indicators except the percentage of drugs prescribed in comprehensive and basic PHCs (<0.0001) (Table 1).

**Table 1:** WHO/INRUD core drug use indicators in selected primary healthcare centers in Lagos State, Nigeria

		Primary Health Centers (PHCs)			
	Optimal value	All (N = 88)	Comprehensive (N = 25)	Basic (N = 63)	
Core drug use indicators		Mean (SD)	Mean (SD)	Mean (SD)	P-value
**Prescribing indicators**					
Average drugs/encounter	1.6-1.8	3.6 (0.9)	3.8 (0.9)	3.6 (0.9)	0.2224
% Generics	100	76.5 (18.5)	75.9 (15.1)	76.7 (19.5)	0.8586
% Antibiotics	20.0-26.8	63.3 (19.1)	64.3 (17.6)	63.0 (19.7)	0.7889
% Injections	13.4-24.1	21.1 (24.1)	27.1 (28.1)	19.3(22.7)	0.2653
% Essential Drugs List (EDL)	100.0	89.2 (2.8)	93.4(2)	87.9 (1.6)	<0.0001
**Patient care indicators**					
Average consultation time (min)	≥ 10	10.5 (6.0)	9.5 (4.4)	11.0 (6.4)	0.1491
Average dispensing time (sec)	≥ 90	244.9 (179.2)	243.2 (124.5)	245.4 (193.1)	0.9516
% Drugs dispensed	100.0	88.8 (10.0)	92.1 (7.3)	87.9 (10.5)	0.0489
% Drugs adequately labeled	100	43.0 (38.3)	40.6 (41.6)	43.8 (37.6)	0.7606
% Adequate knowledge	100	72.4 (38.3)	72.8 (37.2)	72.3 (38.9)	0.9557
**Facility specific indicators**					
% EDL availability	100.0	98.6(12.0)	93.8 (25)	100.0 (0.0)	NA
% Key drugs in stock	100.0	89.8 (3.5)	95.3 (1.8)	88.2 (1.9)	<0.0001*

* significant difference p<0.05

**Patient-care indicators:** for all the PHCs, the average consultation time was 10.5 ± 6.0 minutes. The average dispensing time was 244.9 ± 179.2 seconds. The percentage of patients´ knowledge of the correct dosage in this study was 72.4 ± 38.3%. The difference among the PHCs were not statistically significant for all patient-care indicators (Table 1).

**Facility-specific indicators:** the percentage availability of the EDL copy was 98.6 ± 12% and of key drugs in the stock (Annex 1) as 89.8 ± 3.5%. Among the PHCs, there was a significant difference for percentage availability of key drugs in the stock between comprehensive and basic PHCs (0.0001) (Table 1). The values of Index of Rational Drug Prescribing (IRDP) and Index of Rational Patient-Care Drug Use (IRPCDU) ranked 1 for basic PHC and 2 for comprehensive PHCs. For Index of Rational Facility Specific Drug Use (IRFSDU), both were ranked 1 (Table 2).

**Table 2:** performance indicators for selected primary health centres in Lagos State, Nigeria

	Primary Health Centers (PHCs)		
Performance indicators	All (88)	Comprehensive (25)	Basic (63)
**Prescribing indicators**			
Non-polypharmacy index	0.5	0.5	0.5
Generic name index	0.8	0.8	0.8
Rational antibiotic index	0.4	0.4	0.4
Injection safety index	1.0	0.9	1.0
Index of drugs on Essential Drugs List (EDL)	0.9	0.9	0.9
Index of Rational Drug Prescribing (IRDP)	3.6	3.5	3.6
Rank	1	2	1
**Patient care indicators**			
Consultation time index	1.0	0.9	1.0
Dispensing time index	1.0	1.0	1.0
Dispensed drugs index	0.9	0.9	0.9
Labeled drugs index	0.4	0.4	0.4
Patients' knowledge index	0.7	0.7	0.7
Index of Rational Patient-Care Drug Use (IRPCDU)	4.0	3.9	4.0
Rank	1	2	1
**Facility specific indicators**			
Index of EDL availability	1.0	0.9	1.0
Index of key drugs in stock	0.9	1.0	0.9
Index of Rational Facility Specific Drug Use (IRFSDU)	1.9	1.9	1.9
Rank	1	1	1
**Grand total**			
Index of Rational Drug Use (IRDU)	9.5	9.3	9.5
Rank	1	2	1

## Discussion

**Drug prescribing indicators:** the average number of drugs prescribed at the selected PHCs were more than twice (3.6) the optimal range recommended by the WHO/INRUD (1.6-1.8) [9] indicating practice of probable polypharmacy. This is similar to studies carried out in primary healthcare centres in Southern Asia [12], Africa [6,32] and Nigeria [26]. It is however lower than findings from a study carried out in Shomolu, Lagos State which reported the average drugs prescribed as 4.7 [33]. The medical doctors at the Comprehensive PHCs consult patients with comorbid conditions which may require more than two drugs in most cases. The average number of drugs per encounter prescribed at the basic PHCs deviates from the WHO reference standards (1.6-1.8). Patients´ presentation with comorbid conditions at the time of visit has been identified as a challenge that makes polypharmacy especially in developing countries inevitable. Polypharmacy definition has been recently reviewed and the most reported definition was the numerical definition of five or more medications daily due to multiple illnesses [34]. Rational prescribing is however encouraged as polypharmacy affects treatment outcomes and could lead to adverse drug reactions [7].

This present study showed no significant difference between the comprehensive and basic PHCs surveyed in terms of generic prescribing. It was found that the percentage of generic prescribing prescribed across all the PHCs was 76.5% ± 18.5 which is below the WHO recommended standard (100%). A range of 27-86% of the drugs were prescribed using their generic name in primary health centers in Bangladesh, Kenya and Tanzania [35-37]. In Nigeria, a study carried out in Warri, showed low generic prescribing at the public hospital (54%) [38], similarly a study carried out in Osun State, Nigeria across 20 PHCs in four local government showed an average percentage of drugs prescribed in generic name as 69.81% [15]. It is expected that the percentage is close to the optimal value of 100%. This helps to reduce cost of drug treatment, rationalisation of drug therapy, allows provision of alternatives for choices and affordability [16,24,39,40]. It also helps to maintain a better economic stock control system that is based on reasonably affordable drugs [41]. Findings from this study showed the need to advocate more on generic prescribing at the primary health care centres in Lagos State.

Percentage of prescription with antibiotic was 63.3% for all the centers with no significant difference in prescription pattern at the comprehensive and basic PHCs (P > 0.7889). This is thrice the WHO standard (20-26.8%) [9]. Percentage of encounter with antibiotics is higher in this study than those found in Egypt 36.0 ± 48.04%, China 48.43%, and Pakistan 48.9% [2,7,11]. It was however lower than the study in Kenya 84.8% [37]. In a study carried out to evaluate prescribing pattern in PHCs in Osun State, Nigeria, the percentage of antibiotics per encounter was 50.10% [15]. Inappropriate use of antibiotics results in antimicrobial resistance which is one of the reasons for therapeutic failure. Llor C and Bjerrum L (2014) reported that overprescribing of antibiotics is associated with an increased medicalization of self-limiting conditions and more frequent re-attendance at health facilities. Antibiotic over prescribing is a particular problem in primary care, where viruses cause most infections [42]. Many adverse drug reactions are also as a result of the use of antibiotics [43]. Healthcare workers especially at the level of primary healthcare need continuous medical education on the importance of rational antibiotics prescribing.

This study showed that the percentage of injections prescribed in all PHCs was found to be 21.1 ± 24.1% while 27.1 ± 28.1% was reported at the comprehensive PHCs and 19.3 ± 22.7% for the basic PHCs. The percentage of injections prescribed across all PHCs and at basic PHCs was within the optimal level (13.4-24.1) while the value reported at the comprehensive PHCs did not fall within the optimal range. It may be attributed to the fact that basic PHCs do not stock many injections and doctors are available only at the comprehensive PHCs. Similarly, a study in Kenya showed the injections prescribed were almost within the optimal range at 24.9% [37]. Use of injections above the standard may lead to unnecessary injection related cost such as risk of transmitting potential infections through needle stick injury, titrating of overdose and the cost of its reversal. Oral alternatives have been encouraged to be promoted at all levels of healthcare [44].

Regarding drugs prescribed from the Essential Medicine list (EDL), findings shows that 89.2% of all drugs prescribed was found in the EDL. This is lower than the optimal reference value of 100% and lower than previous studies carried out in regional Africa; Ethiopia (92%) [45] and locally (94%) [15]. It is however higher than findings from a previous study carried out in Ghana, Egypt (69.5%; 81.2%) [2,46] and in Lagos 83.2% [33]. This is comparable to the overall essential medicine list prescribing adherence of 88.0% found in a systematic analysis of prescribing indicators at primary health care centers within the WHO African region [47]. Most of the PHCs in Lagos State operate a Sustainable Drug revolving fund (SDRF) scheme [48]. Most of the medicines prescribed are from SDRF list which is updated monthly to inform prescribers of the available medicines. There is however need for Pharmacists to disseminate a drug bulletin among prescribers monthly to keep them abreast of the available medicines. It is also necessary that the prescribers adhere strictly to the facility drug bulletin. Drug and Therapeutic Committees should be established to optimize rational drug use across the PHCs. The IRDP calculated by adding the index values of all prescribing indicators in this study for comprehensive and basic PHCs was found to be 3.5 and 3.6 respectively. These values were lower than the ideal which is 5. The IRDP values were lower than those reported in Pakistan (3.38 to 4.27) and Ethiopia (4.42-4.46) [6,13] but higher than a study carried out in Sierra Leone (2.6) [10]. The findings from this study shows that prescribing practices should be improved upon.

**Patient-care indicators:** the results of the present study demonstrated that the average consultation time was optimal across the PHCs (10.5 ± 6.0min) and at basic PHCs (11 ± 6.6 min) while the consultation time within the comprehensive centers (9.1 ± 4.4min) was lower than the optimal limit. The reason some comprehensive PHCs reported a value below the optimal time limit may be attributed to high patients turn out. The optimal consultation time recommended is ≥ 10 mins [37] and this is to enable proper history taking, appropriate physical examination and health education. Adequate time with patients will allow feedback from patient, confidence in the treatment plan and expected therapeutic outcome. The dispensing time reported in this study in all the PHCs was 244.9 ± 179.2 secs. The optimal dispensing time according to the WHO/INRUD specification should be ≥ 90 sec. Some previous studies showed average dispensing time were lower than that of the current study, ranging from 38 to 152.3 seconds [6,12,37,49]. A dispensing time < 90 sec is not sufficient according to the WHO criteria which states a pharmacist must spend at least three minutes orientating the patients before handing over the medication [49]. Adequate time is needed for sorting, labelling, patient education on the dosage regimen, precautions including adverse effects of drugs. This may be the reason for longer dispensing time at all the PHCs as the dispensing process will involve counselling patients on use of their medications. About eighty- nine percent (89%) of drugs prescribed was dispensed in all the PHCs while 92.7% of drugs prescribed was dispensed at the comprehensive centres. These values are lower than the optimal value of 100%. It was however higher than previous studies in Nigeria that reported 56.17% [24]. Adequacy of labelling of the drugs prescribed was 43% in all the PHCs which is lower than the recommended value of 100%. It was lower at both comprehensive PHC (40.6%) and basic PHCs (43.8%).

Previous studies in Nigeria, Ethiopia and Kenya reported low values in labelling adequacy ranging from 0% ,17.5% and 22.6% [24,37,50] on medicine packs and drug envelopes. There was no significant difference between the comprehensive and basic PHCs in terms of adequacy of labelling of medicines (p-value 0.7606). Patient´s knowledge of correct dosage is highly beneficial in enhancing patients´ compliance, reduction of drug over use and to prevent adverse effects on patients. About seventy - three percent (73%) of patients seen during the study period had adequate knowledge of the medicines dispensed. The patients at the comprehensive PHCs had better knowledge of their medicines at 72.8% when compared with basic PHCs (72.3%). There was no significant difference between the comprehensive PHCS and the basic PHCs (p-value 0.9557). The reported values from this study is however higher than findings from previous studies (54.7%, 69%, 30%) [37,45,51].

The IRPCDU values shows better performance at the basic PHCs (4.0) when compared with the comprehensive PHCs (3.9). Both types of PHCs showed IRPCDU values lower than the ideal value of 5. The values were higher than that found in studies carried out in Egypt (2.82-3.19) [12] and Pakistan (2.49-3.96) [6]. The labeled drugs index and patient´s knowledge index was 0.4 and 0.7 respectively at both the comprehensive and basic PHCs. These values are lower than the ideal value of 1. The Labelled drug index and the Patients knowledge index reported in the study also shows the need to build the capacities of the dispensers by retraining them on proper labelling of medication packages and drug envelopes. There is also need for the dispensers to further improve on information provided to patients about their medications during counselling.

**Facility-specific indicators:** the study revealed that the percentage availability of EDL in all the PHCs was 98.6 ± 12%. Findings from previous studies in Ethiopia and Pakistan showed the availability was 93.75% and 100% respectively which was higher than findings from this study [6]. However, a review of the rational use of drugs in Nigeria reported the availability of keys drugs as 86.5% [26]. The statistically significant difference in availability of key drugs in stock between the comprehensive and basic PHCs (p value 0.0001) may be because comprehensive PHCs in Lagos State operate a 24 hours service with full complement of staffs including medical doctors and a strong sustainable drug revolving fund [48,52]. The IRFSDU for the index of key drugs in stock showed the comprehensive and basic PHCs reported values of (1) and (0.9) respectively. This goes further to depict that both types of PHCs have good inventory control and availability of key drugs in stock which may be attributed to the Sustainable Drug Revolving Fund Programme in operation at the PHCs [48].

**Limitations of the study:** the study was not designed to investigate the reasons for the irrational prescribing and dispensing practices observed in the study. Future studies will be required to further investigate the reasons for the irrational drug use practices at the PHCs. It was a cross sectional study of primary health facilities which did not include the secondary and tertiary health facilities. The assessment was carried out across of a sample of selected PHCs in Lagos State.

## Conclusion

This study demonstrated irrational drug use practices for some indicators as shown by values reported for the non-polypharmacy index, rational antibiotic index, labelled drugs index and patients' knowledge index in both comprehensive and basic PHCs. There was no significant difference in most of the core drug use indicators between the comprehensive and basic PHCs. There is need to implement interventions aimed at strengthening good prescribing and patient-care practices to ensure rational drug use across the PHCs in Lagos State.

### What is known about this topic

- Irrational prescribing, dispensing and drug use practices exists in many health facilities;
- This is very significant in developing countries;
- The consequence has led to irrational drug use and loss of confidence in health systems.

### What this study adds

- There is irrational drug use practices in Primary Health Centres in Lagos State, Nigeria;
- Patients' knowledge of drugs dispensed is poor and not in line with WHO/INRUD recommendations;
- There is need to implement interventions aimed at strengthening good prescribing and patient-care practices across the PHCs in Lagos State.

## Annex

**Annex 1**: key drugs for testing drug availability (PDF-428KB)
